# Presenteeism, health-related productivity loss, burnout, quality of nursing care, and retention intention among nurses: a structural equation modeling study

**DOI:** 10.3389/fpubh.2026.1962028

**Published:** 2026-10-14

**Authors:** Mengyun Peng, Jiqin Ma, Wenyu Shi, Suyan Chen, Zixuan Zhang, Xiaoling Zhu

**Affiliations:** 1 School of Nursing, Suzhou Medical College, Soochow University, Suzhou, China; 2 Department of Nursing, First Affiliated Hospital of Dali University, Dali, China

**Keywords:** burnout, health-related productivity loss, presenteeism, quality of nursing care, retention intention, structural equation modeling

## Abstract

**Background:**

The interrelationships between presenteeism, health-related productivity loss, burnout, quality of nursing care, and retention intention remain insufficiently understood in nursing populations.

**Objective:**

This study examined the associations between presenteeism, health-related productivity loss, burnout, quality of nursing care, and retention intention.

**Methods:**

A cross-sectional survey was conducted involving 511 nurses. Composite scores from the Work Productivity and Activity Impairment (WPAI) questionnaire, the Maslach Burnout Inventory - Human Services Survey (MBI-HSS), the Quality of Nursing Care Tool, and the Nurse Retention Intention Scale were analyzed using an observed-composite path structural equation modeling (SEM) approach. Indirect effects were estimated using 5,000 bootstrap resamples with percentile-based 95% confidence intervals.

**Results:**

Presenteeism was positively associated with burnout (β = 0.262, *p* < 0.001) and negatively associated with the quality of nursing care (β = −0.250, *p* < 0.001) and retention intention (β = −0.334, *p* < 0.001). Burnout was negatively associated with the quality of nursing care (β = −0.177, *p* < 0.001) and retention intention (β = −0.179, *p* < 0.001). Bootstrap analyses indicated indirect associations between presenteeism and productivity loss and both outcomes through burnout. The model showed an acceptable but imperfect fit: χ^2^ (4) = 13.151, CFI = 0.959, TLI = 0.877, RMSEA = 0.067, and SRMR = 0.043.

**Conclusion:**

Higher presenteeism was statistically associated with lower retention intention and poorer nursing care quality, both directly and indirectly, through burnout. Health-related productivity loss showed indirect associations with both outcomes through burnout.

## Background

Nurses are an indispensable and vital component of healthcare systems worldwide, and their health directly impacts patient safety and the quality of care provided (1). Presenteeism refers to attending work despite a health problem that would reasonably justify taking rest or sick leave, while health-related productivity loss refers to a reduction in work output due to health problems (2, 3). These concepts overlap but are not interchangeable: Presenteeism describes the behavior of working while ill, while health-related productivity loss describes the resulting impairment in work performance. Nurses may continue working because of professional responsibility, staffing shortages, attendance expectations, or concerns regarding burdening their colleagues. Recent evidence links sickness presenteeism in nursing with impaired work functioning, burnout, and missed nursing care (3–5), highlighting its relevance to both nurses’ well-being and the stability of care delivery.

Prior research links working while ill to reduced concentration, impaired work functioning, and missed nursing activities (4). Burnout is also associated with poorer perceived care quality and workforce instability (5, 6). However, the direction and magnitude of these associations may depend on organizational support, workload, and professional commitment. As a result, burnout was identified as a possible statistical mediator, without assuming a causal sequence.

The relationship between presenteeism and retention intention has been inconsistent across studies. Some studies has found lower willingness to remain among nurses who report sickness presenteeism, whereas professional commitment and organizational support may promote retention despite work-related strain (7, 8). In the present study, retention intention is the outcome: A negative coefficient means lower willingness to remain in nursing.

Health-related productivity loss extends beyond presenteeism and may include impaired performance at work and limitations in daily activities (9). Previous studies have shown that presenteeism can directly lead to health-related productivity loss and even increased costs (10). In addition, health-related productivity loss may be statistically related to burnout, but reciprocal or confounding factors could also influence this correlation in cross-sectional data (11). The present model, therefore, estimates associations and indirect effects rather than causal pathways.

Few studies have simultaneously examined presenteeism and health-related productivity loss as distinct predictors of burnout, quality of nursing care, and retention intention within one model. This is important because the behavior of working while ill and the extent of productivity impairment may have different relationships with work outcomes. A conservation of resources (COR) theory proposes that individuals seek to preserve valued resources such as health, energy, time, and emotional capacity (12, 13). Presenteeism and productivity loss may be directly associated with the quality of nursing care and retention intention because they reflect constrained resources at work; they may also show indirect associations through burnout when continuing resource demands coincide with emotional exhaustion and disengagement. By testing these direct and indirect associations simultaneously, this study extends previous nursing research and identifies whether burnout represents a potentially relevant target for workforce support strategies.

Therefore, guided by COR theory, this study examined whether presenteeism and health-related productivity loss were directly associated with the quality of nursing care and retention intention and whether they showed indirect associations with these outcomes through burnout among clinical nurses (Figure 1).

**Figure 1:**
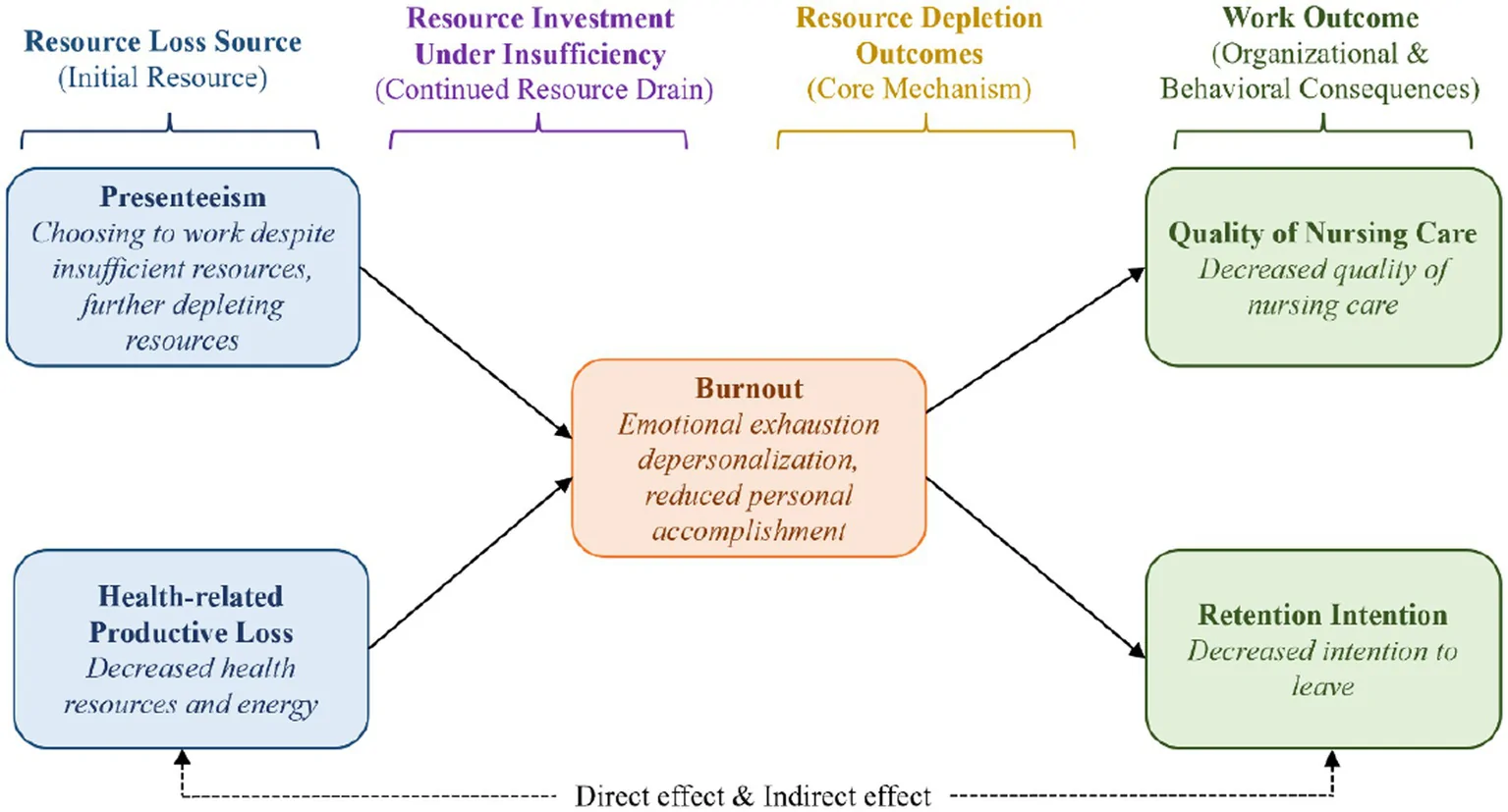
Theoretical framework based on Conservation of Resources (COR) theory.

## Methods

### Study design and participants

This study employed a multi-center, cross-sectional design with convenience sampling. A total of 600 nurses were invited through the nursing departments of five hospitals in Yunnan, Sichuan, and Henan provinces between October and December 2025. In total, 511 valid questionnaires were returned (response rate 85.17%). The five hospitals were selected because they provided access to clinical nursing staff across different hospital settings and were willing to support recruitment. Eligible participants held a valid nursing license, had at least 1 year of direct care experience, and provided consent. Nurses not scheduled during the data collection period, advanced students, and interns were excluded. The electronic questionnaire was distributed by trained nurse coordinators, and each participant could submit only once.

According to Comrey and Lee (14), a sample size of approximately 300 is generally regarded as sufficient for factor analysis. The final analytic sample comprised 511 nurses. A prospective *a priori* structural equation modeling (SEM) power calculation was not documented at the time of data collection. Therefore, we do not retrospectively label the existing *post hoc* calculation as an *a priori* calculation. The sample size was nevertheless substantially larger than common minimum recommendations for an observed path model with the present number of predictors.

### Measurements

#### Sociodemographic characteristics

A self-designed sociodemographic characteristics questionnaire was initially developed, including age, sex, working duration, marital status, number of children, department, type of hospital, hospital level, professional title, position, highest education level, employment type, number of overtime shifts in the past month, and number of night shifts per month.

#### Stanford Presenteeism Scale-6

The Stanford Presenteeism Scale-6 (SPS-6) was used to assess sickness presenteeism during the previous month (15, 16). The scale contains six items rated on a 5-point Likert scale ranging from 1 (strongly disagree) to 5 (strongly agree). Items 1–4 are scored in their original direction, whereas the positively worded items 5 and 6 are reverse-coded using the formula 6 minus the item score, ensuring that all items are scored in the same direction. The six item scores are summed to produce a total score ranging from 6 to 30. Higher scores indicate greater sickness presenteeism and greater health-related impairment while working, whereas lower scores indicate a better ability to maintain work functioning despite health problems. The Cronbach’s alpha coefficients for the scale’s dimensions ranged from 0.76 to 0.90. In the present study, the Cronbach’s alpha coefficients ranged from 0.745 to 0.883.

#### Health-related productivity loss

The Work Productivity and Activity Impairment - General Health (WPAI-GH) questionnaire was used to assess health-related productivity loss during the previous 7 days (17). Its six items assess employment status (Q1), health-related absenteeism (Q2), absence for other reasons (Q3), hours actually worked (Q4), health-related impairment while working (Q5), and impairment in regular activities (Q6). The primary outcome was overall work productivity impairment, calculated among currently employed nurses using the following formula: [Q2/(Q2 + Q4) + {1 − Q2/(Q2 + Q4)} × (Q5/10)] × 100. Scores range from 0 to 100%, with higher percentages indicating greater productivity loss. Participants who were not employed or had Q2 + Q4 = 0 were coded as missing. This deterministically calculated percentage was analyzed as an observed continuous variable rather than a latent construct. As its components form the index rather than reflect a single latent trait, Cronbach’s alpha was not calculated for the WPAI outcome.

#### Maslach Burnout Inventory—Human Services Survey (MBI-HSS)

The Maslach Burnout Inventory - Human Services Survey (MBI-HSS) was used to assess occupational burnout (18). The 22 items cover emotional exhaustion, depersonalization, and personal accomplishment and are rated on a 7-point scale ranging from 0 (never) to 6 (every day). Dimension scores and the overall score were calculated according to the validated scoring protocol, with the personal accomplishment component coded so that higher overall scores represented greater burnout. The possible score and direction of each dimension were checked before computing the composite score. The Cronbach’s alpha coefficient for the scale was 0.93. In this study, the Cronbach’s alpha coefficient was 0.85.

#### Quality of nursing care

The Quality of Nursing Care Tool is a 10-item self-assessment completed by nurses to assess individual-level perceived nursing care performance (19). It is not an objective measure of patient safety or an organization-level outcome. It includes 10 items: Nursing care plans, specialized nursing care, ward management, catheter care, health education, implementation of handover procedures, implementation of verification procedures, implementation of tiered nursing care, implementation of safety measures, and implementation of basic nursing care. Each item is scored on a scale of 1 to 10. A higher score indicates better nursing quality. The Cronbach’s alpha coefficient for the scale was 0.862. In the present study, the Cronbach’s alpha coefficient was 0.915.

#### Retention intention

The Nurse Retention Intention Scale was used to assess nurses’ willingness to remain in their current nursing position (20). The scale contains six items rated on a 5-point scale ranging from 1 (strongly disagree) to 5 (strongly agree). Items were coded in the direction of retention and summed to produce a total score ranging from 6 to 30, with higher scores indicating stronger retention intention. The Cronbach’s alpha coefficients for the scale’s dimensions ranged from 0.78 to 0.86. In the present study, the Cronbach’s alpha coefficients ranged from 0.79 to 0.88.

### Data collection

This study administered electronic questionnaires using the Wenjuanxing platform, which is currently the most widely used online data collection platform in China. To prevent duplicate responses, the questionnaire was configured to permit only one submission per unique mobile phone number or IP address. All items were designated as mandatory, ensuring that incomplete questionnaires could not be submitted. Prior to the formal survey, the research team member contacted the directors of nursing at the five hospitals and provided them with a detailed explanation of the study’s objectives, procedures, and significance. Following their approval, the team delivered comprehensive training materials to the five nurses responsible for distributing the electronic questionnaires. These materials primarily contained detailed instructions regarding precautions and procedures for completing the questionnaires. Subsequently, the research team members verified the data in the backend system to confirm the completeness and reliability of the responses.

### Data analysis

Statistical analyses were conducted using IBM SPSS Statistics 20.0 and AMOS 20.0. Descriptive statistics were used to summarize participant characteristics and study variables, and Pearson’s *r* was used because the five analyzed composite scores were continuous. Presenteeism, health-related productivity loss, burnout, quality of nursing care, and retention intention were treated as observed continuous composite variables. The prespecified path model included burnout as a mediator: Presenteeism and productivity loss predicted burnout, and presenteeism, productivity loss, and burnout predicted quality of nursing care and retention intention. Maximum likelihood estimation was used, and the model had 4 degrees of freedom. All questionnaire items were mandatory in the electronic survey, and the final dataset contained no missing composite scores. Therefore, no imputation was performed. Variable distributions were screened before modeling, and the productivity loss score showed evidence of non-normality. Accordingly, indirect effect inference was based on 5,000 nonparametric bootstrap resamples with percentile-based 95% confidence intervals. Model fit was evaluated jointly using χ^2^, χ^2^/*df*, CFI, TLI, RMSEA with its confidence interval, and SRMR. Values of CFI and TLI approximately ≥0.90, RMSEA ≤0.08, and SRMR ≤0.08 were considered indicative of acceptable approximate fit (15). No modification indices, residual correlations, or data-driven pathways were added.

## Results

### Sociodemographic characteristics

This study included 511 nurses, with an average age of 33.29 ± 7.15 years. Overall, 90.8% of the participants were female, and 89.8% held a bachelor’s degree. Other details are provided in Table 1.

**Table 1:** Sociodemographic characteristics of participants (*n* = 511).

Characteristics	*n*	%	Mean	*SD*
Age (years)			33.29	7.15
Sex				
Male	47	9.2		
Female	464	90.8		
Working duration			10.53	7.61
Marital status				
Married	353	69.1		
Single	158	30.9		
Number of children				
0	196	38.4		
1	192	37.6		
2	121	23.7		
>2	2	0.4		
Department				
Department of Internal Medicine	159	31.1		
Department of Surgery	163	31.9		
Emergency Department	22	4.3		
ICU	83	16.2		
Operating Room	14	2.7		
Department of Obstetrics and Gynecology	9	1.8		
Department of Pediatrics	15	2.9		
Department of Psychiatry	3	0.6		
Outpatient Department	8	1.6		
Others	35	6.8		
Type of hospital				
General hospital	479	93.7		
Specialist hospital	32	6.3		
Level of the hospital				
Grade A Tertiary hospital	478	93.5		
Grade B Tertiary hospital and below	33	6.5		
Professional title				
Nurse	102	20.0		
Nurse practitioner	147	28.8		
Supervisor nurse	215	42.1		
Associate chief nurse and above	47	9.2		
Position				
Clinical Nurse	453	88.6		
Head Nurse and Above	58	11.4		
Highest education level				
Associate Degree	12	2.3		
Bachelor’s Degree	459	89.8		
Master’s Degree or Above	40	7.8		
Employment type				
Contract-based employment	395	77.3		
Permanent staff	116	22.7		
Number of overtime shifts in the past month				
0	205	40.1		
1–3	207	40.5		
>3	99	19.4		
Number of night shifts per month				
0	119	23.3		
1–4	177	34.6		
5–8	188	36.8		
>8	27	5.3		

### Scores for presenteeism, health-related productivity loss, burnout, quality of nursing care, and retention intention among nurses

The average scores for presenteeism, health-related productivity loss, burnout, quality of nursing care, and retention intention were 14.90 (SD = 4.55), 67.60% (SD = 13.42%), 83.69 (SD = 20.20), 82.16 (SD = 15.46), and 22.17 (SD = 4.35), respectively. Detailed results are presented in Table 2.

**Table 2:** Descriptive statistics for variables (*n* = 511).

Variables	Mean	SD
Presenteeism	14.90	4.55
Work constraints	9.47	4.21
Work outcomes	5.43	2.33
Health-related productivity loss	67.6 (%)	13.42 (%)
Burnout	83.69	20.20
Quality of nursing care	82.16	15.46
Retention Intention	22.17	4.35

### Correlations between variables

Pearson correlation analyses showed that retention intention was negatively correlated with presenteeism (*r* = −0.393, *p* < 0.001), productivity loss (*r* = −0.136, *p* = 0.002), and burnout (*r* = −0.275, *p* < 0.001) and positively correlated with quality of nursing care (*r* = 0.280, *p* < 0.001). Quality of nursing care was negatively correlated with presenteeism (*r* = −0.296, *p* < 0.001), productivity loss (*r* = −0.039, *p* = 0.374), and burnout (*r* = −0.243, *p* < 0.001). The complete correlation matrix is shown in Table 3.

**Table 3:** Correlations among variables (*n* = 511).

Variables	Presenteeism	Health-related productivity loss	Burnout	Quality of nursing care	Retention Intention
Health-related productivity loss	0.153**	—	—	—	—
Burnout	0.268**	0.084	—	—	—
Quality of nursing care	−0.269**	−0.039	−0.243**	—	—
Retention Intention	−0.393**	−0.136**	−0.275**	0.280**	—

*p < 0.05; **p < 0.01. Indirect effects are based on 5,000 bootstrap resamples.

### Structural equation modeling

Table 4 presents the model results. The final observed-composite SEM showed acceptable but imperfect fit (Table 5): χ^2^(4) = 13.151, *p* = 0.011, χ^2^/df = 3.288, CFI = 0.959, TLI = 0.877, RMSEA = 0.067, and SRMR = 0.043. Presenteeism was associated with burnout, quality of nursing care, and retention intention. Burnout was associated with both quality of nursing care and retention intention. Figure 2 presents the final model.

**Table 4:** Standardized direct, indirect, and total effects in the final model.

Pathways	Direct effect	Indirect effect	Total effect
Presenteeism → burnout → quality of nursing care	−0.250***	−0.045***	−0.295
Presenteeism → burnout → retention intention	−0.334***	−0.045***	−0.379
Health-related productivity loss → burnout → quality of nursing care	0.014	−0.013**	0.001
Health-related productivity loss → burnout → retention intention	−0.070	−0.013**	−0.083

*p < 0.05; **p < 0.01; ***p < 0.001. Indirect effects are based on 5,000 bootstrap resamples.

**Table 5:** Fit indices for the final observed-composite SEM.

Fit index	*χ*^2^ (*p*)	df	*χ*^2^/df	CFI	TLI	RMSEA	95% CI	SRMR
Final model	13.151 (0.011)	4	3.288	0.959	0.877	0.067	0.029–0.109	0.043

**Figure 2:**
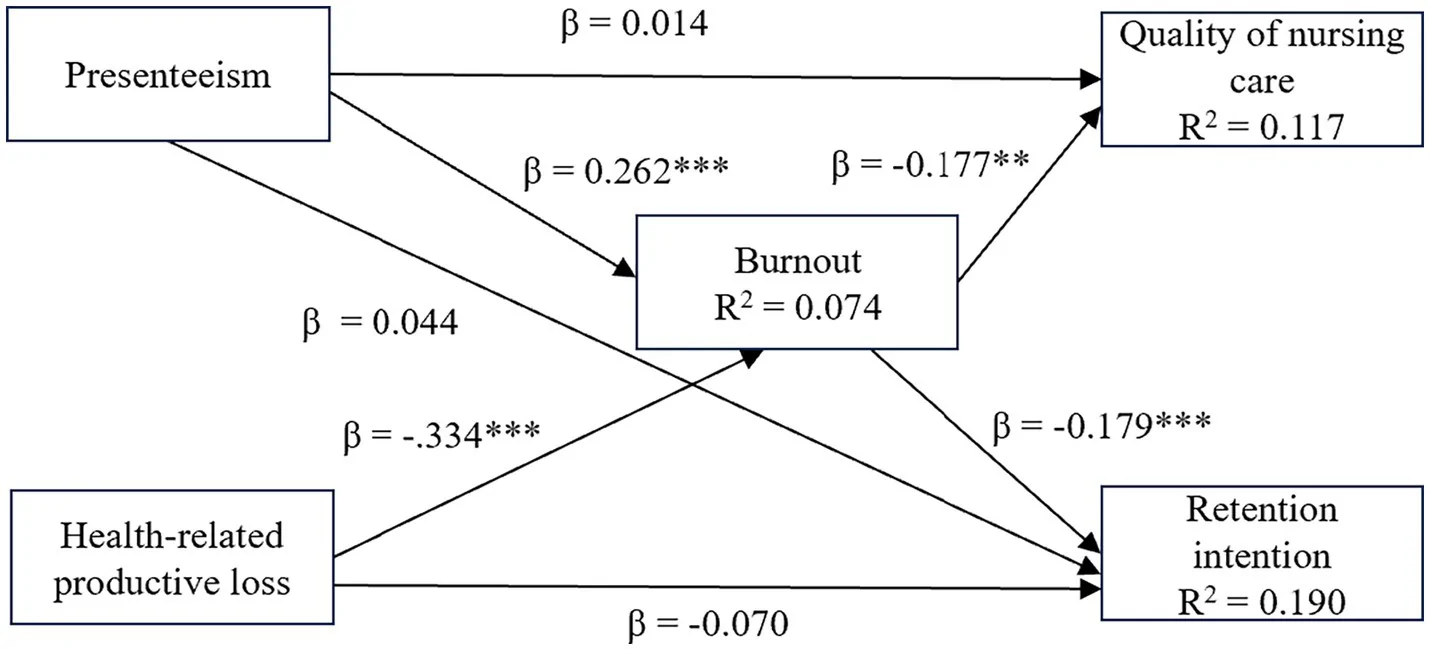
Final observed-composite path model with standardized β coefficients. Solid arrows: *p* < 0.05; dashed arrows: *p* ≥ 0.05. Values are standardized β coefficients. Indirect effects were estimated using 5,000 bootstrap samples. **p < 0.01; ***p < 0.001.

## Discussion

This study aimed to test a hypothetical model examining whether presenteeism and health-related productivity loss influence the quality of nursing care and retention intention, both directly and indirectly through burnout, among clinical nurses. Using structural equation modeling, several key findings emerged. First, higher presenteeism was associated with greater burnout, poorer self-rated care quality, and lower retention intention. Second, greater burnout was associated with poorer care quality and lower retention intention. Third, although the direct pathways from health-related productivity loss to burnout, quality of nursing care, and retention intention were not statistically significant, bootstrap analyses identified small indirect associations with both outcomes through burnout. The final model showed mixed fit indices and explained 7.4% of the variance in burnout, 11.7% in care quality, and 19.0% in retention intention. These findings make an important theoretical contribution, indicating that, while presenteeism and health-related productivity losses may reduce nurses’ retention intention, they may not be risk factors leading to a decline in the quality of nursing care. Therefore, nursing managers and administrators should adopt multi-level strategies to simultaneously address presenteeism, health-related productivity loss, and burnout to effectively enhance nursing quality and stabilize the nursing workforce (Table 6).

**Table 6:** Bootstrap indirect effects based on 5,000 resamples.

Indirect pathway	B indirect	Bootstrap SE	95% CI	*p*
Presenteeism → burnout → quality of nursing care	−0.045	0.015	−0.077 to −0.017	< 0.001
Presenteeism → burnout → retention intention	−0.045	0.014	−0.074 to −0.021	<0.001
Health-related productivity loss → burnout → quality of nursing care	−0.013	0.010	−0.039 to −0.003	0.002
Health-related productivity loss → burnout → retention intention	−0.013	0.010	−0.038 to −0.003	0.002

The study demonstrated that presenteeism was related to nurses’ intention to leave through two pathways: Directly and indirectly through burnout. This finding aligns with the findings of a Japanese study that focused on middle-aged nurses (21). The direct effect suggests that working while ill impairs physical condition and concentration, which reduces work efficiency and job satisfaction over time and ultimately strengthens the desire to leave (7). The indirect pathway operates through burnout. Previous studies have shown that repeated presenteeism gradually depletes emotional resources, leading to increased emotional exhaustion (22). As burnout intensifies, nurses become more detached from their work and less committed to the organization, which, in turn, may increase their intention to leave (23). These two pathways indicate that presenteeism is not a harmless sign of dedication, but a risk factor for nurses’ intention to leave. This finding has practical implications for workforce stability and should be considered in retention strategies. However, the cross-sectional data cannot determine whether presenteeism precedes burnout or lower retention intention, and workload, organizational support, and professional commitment may also contribute to the observed associations.

Presenteeism was negatively associated with the quality of nursing care, both directly and indirectly through burnout. The direct pathway suggests that when nurses experience health problems, their perceived ability to meet work demands may decline, which may generate frustration and a sense of inadequacy. Over time, this perceived decline in performance may weaken their attachment to the job and increase their intention to leave (24). The indirect pathway through burnout reflects a more gradual process (25). Persistent productivity loss depletes emotional and physical resources, contributing to cumulative exhaustion (26). As burnout intensifies, nurses become emotionally detached from their work and lose professional meaning, further reinforcing their intention to leave (27, 28). These two pathways are not mutually exclusive but may operate simultaneously, amplifying the overall effect. This pattern indicates that health-related productivity loss is not merely a performance issue but also a risk factor for workforce instability. Therefore, interventions aimed at reducing turnover should consider both early identification of productivity loss and management of burnout, rather than focusing on either aspect in isolation. Importantly, care quality was self-rated rather than objectively observed, so longitudinal studies using independent measures of care processes and outcomes are needed.

Productivity loss was not independently associated with retention intention in the direct pathway, but its indirect association through burnout was statistically supported. This pattern suggests that burnout may partially account for the relationship between perceived productivity loss and willingness to remain. Since all variables were measured at one time point, reciprocal and confounded explanations remain possible.

### Implications for clinical practices and management

These findings have substantial practical implications for healthcare administrators and nursing managers. First, administrators and managers should move beyond evaluating health solely through presenteeism metrics. Presenteeism represents a hidden challenge: Nurses who work while ill are physically present, yet their physical distress and the associated risks often go undetected until clinical errors occur. Accordingly, managers should reshape nurses’ attitudes toward sick leave, eliminate the stigmatization of legitimate sick leave, and avoid punitive attendance policies, thereby cultivating a culture of healthy attendance. Second, staffing reserve mechanisms should be established, for example, creating a local floating pool of cross-trained nurses, so that nurse managers can adapt seamlessly when nurses require sick leave, thereby mitigating colleagues’ feelings of guilt. Third, given that burnout serves as the mediating pathway through which both presenteeism and health-related productivity loss influence nurses’ retention intention and quality of nursing care, psychological health screening, resilience training, and peer support programs should be integrated into nursing professional development and retention strategies.

### Limitations

Several limitations should be acknowledged. First, the cross-sectional design prevents temporal or causal conclusions and does not establish that burnout mediates a causal process. Second, convenience sampling from five hospitals across three provinces may have introduced selection bias and limits the generalizability of the findings to nurses working in other hospital levels, regions, specialties, or healthcare systems. Third, all major variables were self-reported at one time point, questionnaires were distributed through nursing departments, and common-method and social-desirability bias were not formally assessed. Fourth, a prospective, SEM-specific power analysis was not documented before data collection; the achieved sample of 511 does not replace a model-specific *a priori* analysis, particularly for small indirect effects. Fifth, productivity loss was non-normally distributed, and the model showed mixed fit indices; the small indirect effects should therefore be replicated. Future research should use prospective, simulation-based sample-size planning, probability or broader multi-site sampling, longitudinal designs, and objective measures of productivity and care quality.

## Conclusion

In conclusion, this study found that higher presenteeism was associated with greater burnout, poorer quality of nursing care, and lower retention intention. Burnout showed statistically supported indirect associations linking both presenteeism and productivity loss to care quality and retention intention. These cross-sectional findings highlight the importance of addressing presenteeism and burnout in workforce support strategies but do not establish causal effects. Healthcare administrators must recognize that requiring sick nurses to continue working, although it may appear to maintain staffing levels, can severely undermine nurses’ well-being. Therefore, targeted interventions designed to reduce presenteeism and health-related productivity loss and alleviate burnout are essential for supporting the long-term sustainability of the nursing workforce.

## Data Availability

The original contributions presented in the study are included in the article/supplementary material, further inquiries can be directed to the corresponding author.
